# Prevalence of healthcare-seeking behavior among caregivers for common childhood illness in Ethiopia using PMA 2021–2023 data a multilevel analysis

**DOI:** 10.1038/s41598-026-45714-8

**Published:** 2026-04-02

**Authors:** Adisu Meles Kabtyimer, Awoke Keleb, Anmut Endalkachew Bezie, Gosa Mankelkl, Altaseb Beyene Kassaw, Halid Worku Jemil

**Affiliations:** 1 Department of Epidemiology and Biostatistics, School of Public Health, College of Medicine and Health Science, Wollo University, Dessie, Ethiopia; 2 Department of Environmental Health, College of Medicine and Health Sciences, Wollo University, Dessie, Ethiopia; 3 Department of Occupational Health and Safety, College of Medicine and Health Science, Wollo University, Dessie, Ethiopia; 4 Department of Biomedical Science, College of Medicine and Health Science, Wollo University, Dessie, Ethiopia; 5 Department of Health Informatics, College of Medicine and Health Science, Wollo University, Dessie, Ethiopia

**Keywords:** Diseases, Health care, Medical research, Risk factors

## Abstract

Healthcare-seeking behavior refers to the actions taken by caregivers to seek medical care for illness. Globally, the failure to seek timely care for common childhood illnesses particularly diarrhea, fever, and cough remain a major contributor to under-five mortality. Timely health care-seeking behavior of caregivers plays a critical role in reducing preventable deaths. However, evidence on individual- and community-level factors influencing care-seeking remains limited in Ethiopia so this study aims to determine prevalence and factors associated with healthcare-seeking behavior for common childhood illness. A secondary data analysis was conducted using the Performance Monitoring for Action (PMA) Ethiopia 2021–2023 Cohort II survey. A total weighted sample of 826 caregivers of under-five children with fever, cough, or diarrhea included. Data preparation involved merging, cleaning, recoding, and management of missing data through multiple imputation, with five imputations and ten iterations, following Rubin’s rules for pooling estimates. Descriptive statistics were computed, and a multilevel logistic regression model was applied to account for the hierarchical structure of the data. Both individual- and community-level factors were examined, and variables with a *p* value < 0.05 were considered statistically significant. Of the 826 weighted caregivers the prevalence of health-seeking behavior among caregivers was 39.6% (95% CI 35.8–43.3%). Healthcare-seeking increased with the number of reported illnesses, ranging from 36.3% for one illness to 54.6% for three illnesses. In the final multivariable multilevel analysis, Mothers who received ANC from health extension workers were 46% less likely to seek care (AOR = 0.54; 95% CI 0.33–0.88), while those who delivered in a health facility were 1.64 times more likely (AOR = 1.64; 95% CI 1.01–2.69). Caregivers whose children had two illnesses were 3.40 times more likely to seek healthcare (95% CI 1.92–6.01), and those with three illnesses were 1.55 times more likely (95% CI 1.06–2.07). Caregivers from richer households had higher odds of seeking healthcare (AOR = 2.10; 95% CI 1.09–4.04). Health-seeking behavior for childhood illnesses in Ethiopia remains low, with fewer than two in five caregivers seeking healthcare. Both individual- and household-level factors were important determinants, highlighting the need to improve maternal health service utilization, strengthen health extension service quality, and address socioeconomic disparities.

## Introduction

Healthcare-seeking behavior refers to the actions taken by caregivers to seek medical care for illness^1^. Globally, the failure to seek timely care for common childhood illnesses—particularly diarrhea, fever, and cough—remains a major contributor to under-five mortality. These symptoms often signal severe illnesses such as diarrhea, pneumonia, and malaria, which collectively account for an estimated 5 million deaths among children under five each year^2^. Sub-Saharan Africa alone accounts for more than 54% of these deaths^3^.

Common childhood illnesses refer to a group of frequently occurring acute conditions among children under five years of age that substantially contribute to childhood morbidity and mortality, particularly in low- and middle-income countries. These illnesses commonly include acute respiratory infection (ARI), fever, and diarrhoea, as reported within the two weeks preceding data collection, and are widely used indicators of childhood morbidity in population-based surveys. Prompt healthcare-seeking for children presenting with these symptoms is therefore critical for early diagnosis, appropriate treatment, and the prevention of severe complications and death^4,5^.

Within Sub-Saharan Africa, diarrhea, acute respiratory infections (ARI), and fever-related illnesses remain leading causes of child morbidity and mortality. However, healthcare-seeking for these conditions is often inadequate. Cross-country data from low- and middle-income countries show that approximately 63% of children with diarrhea and 70% with ARI are taken to a health provider^6^. In Ethiopia, despite improvements since 2000, care-seeking rates remain far below these averages: in 2016, only about 44% of children with diarrhea, 36% with fever, and 29% with ARI symptoms were taken to a health facility^7^.

Several factors influence healthcare-seeking behavior at the individual and community levels. Studies have shown that maternal education, household wealth, ANC follow up, Childs age ,family size, male child and exposure to media positively influence timely care-seeking, while distance to health facilities, rural residence, and cultural reliance on traditional remedies are significant barriers^8–14^.

The consequences of poor healthcare-seeking behavior are multifaceted. For children, untreated illnesses result in avoidable deaths and long-term developmental impairments globally in 2023 alone, roughly 13,100 under-five deaths occurred every day, largely due to preventable child deaths^15^. For caregivers, especially mothers, the emotional and financial burden can be severe. At the national level, low healthcare utilization contributes to economic losses due to lower productivity and increased healthcare costs, and social inequities are reinforced by disparities in access to essential services^16^.

Ethiopia has taken several steps to improve child health, including the Health Extension Program and the implementation of Integrated Management of Childhood Illnesses (IMCI). While these programs have contributed to significant improvements—such as a notable reduction in under-five mortality since 2000—healthcare-seeking rates remain low in many rural and underserved areas^17,18^.

Despite these efforts, important gaps remain. There are gaps about how individual and community-level factors interact to influence healthcare-seeking across all three illnesses (diarrhea, fever, cough). Methodological gaps persist due to the use of single-level statistical approaches that fail to account for the nested nature of health determinants. Contextual gaps are evident, as most existing studies in Ethiopia either focus on single diseases nationally or all three illnesses in limited geographic areas^8,9,19–21^.

Addressing these gaps is urgent. Applying a mixed effect modeling approach can provide a more nuanced understanding of the magnitude and determinants of healthcare-seeking behavior. This evidence can support the development of targeted equity-focused health interventions to reduce child mortality and meet Ethiopia’s commitments to global health goals.so the aim of this study is to determine the prevalence and individual and community-level determinants of caregiver’s healthcare-seeking behavior for common childhood illness in Ethiopia.

## Methodology

### Data source and study area

This study is based on data from the Performance Monitoring for Action (PMA) Ethiopia Panel Cohort 2. The PMA Ethiopia project is implemented by Addis Ababa University in collaboration with the Bill & Melinda Gates Institute for Population and Reproductive Health at Johns Hopkins University. The dataset is publicly available through the Johns Hopkins data repository without any registration.

The study draws on data from Oromia and Amhara regions, the former Southern Nations, Nationalities and Peoples (SNNP) region—specifically from the South Ethiopia, Central Ethiopia, and Southwest Ethiopia regions—and Addis Ababa city; data from Sidama Region were not included^22^. Together, these regions account for 79.5% of Ethiopia’s population and are characterized mainly by urban and agricultural communities. According to the 2023 population estimates from the Ethiopian Statistical Service, these regions are home to 85,286,208 people, of whom 42,478,425 are men and 42,807,783 are women^23^. In Ethiopia, there are an estimated 32,1280,39 reproductive age women and 19,066,529 children under-five in 2023, of these, more than 76% are found in the regions included in this study. The country has a three-tier healthcare delivery system and more than 353 public hospitals, 3706 public health centers and 17,561 health posts. In addition to the public health facilities, there are also more than 5000 other categories of health facilities such as other-government health facilities, private for profit and private-not-for profit health facilities that provide preventive and curative health services.

### Study design and period

The PMA employed a different method to determine study participants. It initially used a cross-sectional study design at baseline and then used a community-based longitudinal study design after they were included in the cohort. The data were collected at four different time points. The baseline data was collected between November 2021 and January 2022. The 6-week data collection was conducted from November 2021 to October 2022. The 6-month data collection was conducted from March 2022 to April 2023. The 1-year follow-up survey was carried out between September 2022 and September 2023.

However, the present study is a secondary data analysis that utilized only the 1-year follow-up dataset of the PMA Ethiopia 2021–2023 Cohort II survey. Although the original PMA study employed a longitudinal cohort design with multiple follow-up points, our analysis was restricted to data collected between September 2022 and September 2023. Therefore, this study is analytically cross-sectional in nature, based solely on the one-year follow-up survey data.

### Source population

All mother–child pairs reside in the Oromiya, Amhara and SNNP 1 regions and Addis Ababa city.

### Study population

The analysis included all caregivers who participated in the year one follow-up survey and reported that their child had experienced diarrhea, fever, or cough in the two weeks preceding the survey.

### Eligibility criteria

#### Inclusion criteria

All mother–child pairs reside in selected enumeration areas of study regions and have child of at least one of the three illness during 2 weeks prior to the 1-year survey.

#### Exclusion criteria

Mother–child pairs who did not participate in the final (one-year) follow-up were excluded. Furthermore, caregivers whose child did not experience any of the three target symptoms during the two weeks preceding the one-year survey were excluded from the analysis.

### Sampling procedure and sample size

The sample for the PMA-ET Panel Cohort 2 Survey was selected using a two-stage cluster sampling design, consistent with the PMA Ethiopia panel methodology and adapted to contextual and administrative changes occurring between Cohort 1 and Cohort 2.

In the first stage, enumeration areas (EAs) for Cohort 2 were drawn from the original PMA Ethiopia panel EAs selected for Cohort 1, which had been sampled from the Central Statistical Agency (CSA) master sampling frame. For Cohort 1, a total of 217 EAs were selected using probability proportional to size (PPS) sampling, where EA selection probability was proportional to the number of households in each EA, as described in the PMA-Ethiopia protocol^24^.

The original allocation of the 217 EAs across regions was based on anticipated fertility levels and the need to produce regionally representative estimates of modern contraceptive prevalence rate (mCPR) with a 5% margin of error, using prior PMA2020 data to inform assumptions on indicator prevalence, design effect, and non-response. These 217 EAs also constituted the sampling frame for the longitudinal panel, allowing the same clusters to be used for both cross-sectional and panel components^24^.

For Cohort 2, the EA sample size was reduced from 217 to 162 EAs due to administrative and security-related changes rather than a revision of statistical power assumptions. Data collection was undertaken only in the original EAs located in Addis Ababa, Amhara, and Oromia. EAs located in Tigray and Afar were excluded due to insecurity, and eight EAs originally selected within the former Southern Nations, Nationalities, and Peoples’ Region (SNNPR) were removed following the establishment of the Sidama Regional State in 2020. As a result, SNNPR was no longer included as a distinct sampling domain in Cohort 2, and the final EA sample comprised 162 EAs, which was deemed adequate for detecting a minimum difference of 5% between comparison groups for the majority of RMNH indicators within the remaining regions^22^.

In the second stage, households within each selected EA were systematically screened to identify eligible women. Eligibility criteria included women aged 15–49 years who were pregnant or who were within 0–9 weeks postpartum at baseline. This eligibility window was selected to align with the panel’s objective of measuring early postnatal outcomes and longitudinal changes in RMNH service utilization. All eligible women identified within sampled households were invited to participate, provided they gave informed consent. Women who did not meet eligibility criteria or declined participation were excluded.

The expected sample size for the original PMA Ethiopia panel was derived from assumptions that approximately 10% of women aged 15–49 years would be pregnant or within six weeks postpartum, requiring screening of approximately 25,000–30,000 women across the 217 selected EAs and yielding an anticipated panel enrollment of approximately 2800 women. Because Cohort 2 covered fewer regions and a reduced number of EAs (162 vs. 217), a smaller enrolled sample size was anticipated without compromising the study’s analytical objectives^22,24^.

For the present secondary analysis, only data from the 12-month follow-up survey (n = 1990) were used. From this dataset, the analytic sample was restricted to caregivers of children who experienced at least one of the three common childhood illnesses—fever, diarrhea, or cough—during the 2-week period preceding the survey. After applying these eligibility criteria, a total of 826 children constituted the final analytic sample for this study.

### Outcome variable

The dependent variable was healthcare-seeking behavior, defined as whether the caregiver sought care for the child’s recent symptoms namely for fever, cough and diarrhea. The outcome variable was coded as: 0 = No (if care was not sought from any of the three symptoms), 1 = Yes (if care was sought for any of the three symptom).

### Independent variables

Individual level variables include Child’s sex, maternal age and education, household wealth tertile, and place of delivery.

Community level variable includes place of residence, region, and proportion of educated women in the EA. These were derived by aggregating individual-level data at the EA level.

### Data preparation and management

From each of the four datasets, variables relevant to the study objectives were identified, extracted, and merged into a single dataset for further analysis. The selected variables were carefully cleaned.

Several variables were derived to capture key constructs. Wealth status was generated by recoding the original five wealth quintiles into three broader categories: poor (lowest and lower quintiles), middle (middle quintile), and rich (higher and highest quintiles)^21^. For women’s empowerment, nine indicators were combined; negatively worded items were reverse-coded prior to aggregation.

Respectful maternity care (RMC) was measured using 14 standardized items available in the dataset, which are aligned with the respectful maternity care framework promoted by the World Health Organization. These items reflect key domains of dignity, communication, consent, emotional support, privacy, and autonomy during childbirth. The variables included: (1) being treated with respect, (2) being spoken to politely, (3) having procedures explained, (4) being asked for consent before examinations, (5) having medications explained, (6) being allowed to ask questions, (7) being involved in decision-making, (8) being allowed a preferred birth position where possible, (9) receiving adequate attention from providers, (10) being encouraged to express concerns, (11) feeling that the best possible care was provided, (12) having privacy maintained during examinations and delivery, (13) being addressed appropriately, and (14) being allowed a birth companion of choice. Data were originally collected by at baseline and at a six-week follow-up. In this secondary analysis, missing baseline responses were replaced with corresponding follow-up responses for the same item to reduce information loss. The total sample consisted of 827 women. Reliability testing indicated strong internal consistency for RMC (Cronbach’s α = 0.899) and acceptable reliability for empowerment (α = 0.678). Exploratory factor analysis (EFA) confirmed one-dimensionality for both constructs, as only the first factor had an eigenvalue greater than one (empowerment = 2.75; RMC = 5.77), with subsequent factors dropping below one. Factor scores were extracted, standardized, and dichotomized into “high” and “low” categories using mean splits.

Community-level variables were created by aggregating individual-level data within clusters. For example, community literacy was derived from maternal education distributions, while community residence was generated from the original residence variable. These aggregated measures were included to capture contextual influences on health-seeking behavior.

#### Missing data management

To address missing data, a customized multiple imputation procedure was conducted in R (version 4.4.2) using mice package. The method was tailored to variable type, and five imputations with ten iterations each were performed. Across the dataset, the overall proportion of missing data was 3.3%, with 45.5% of participants (n = 376) having complete information. The highest levels of missingness were observed in respectful maternity care (36.1%) and pregnancy intention (25.9%), while variables such as intimate partner violence (16.0%), postnatal care within six weeks (4.8%), maternal age category (2.3%), antenatal care by health extension workers (0.4%), and place of delivery (0.8%) had lower levels. After imputation, 13 variables were retained for the main analysis.

The analysis followed Rubin’s rules, which involve three steps: (1) imputing missing values to generate multiple complete datasets, (2) conducting the same analysis on each imputed dataset, and (3) pooling estimates to obtain final coefficients and standard errors that reflect both within- and between-imputation variability^25^.

#### Statistical analysis

Multilevel modeling was applied to identify factors associated with caregiver health-seeking behavior for common childhood illnesses, accounting for the hierarchical structure of the data. Weighted samples (n = 826) were used to estimate prevalence. STATA version 17 was employed for data cleaning and survey weighting, while R version 4.4.2 was used for missing data management, imputation, and multilevel modeling.

The intraclass correlation coefficient (ICC) was used to determine whether significant clustering was present. The following formula was used to calculate the ICC:

$${\mathrm{ICC}} = \, \sigma {\mathrm{u2}}/ \, \left( {\sigma {\text{u2 }} + \, \left( {\pi {2}/{3}} \right)} \right)$$

The null model indicated significant between-cluster variability in health-seeking behavior (variance = 1.037, p < 0.001). The intraclass correlation coefficient (ICC) was 0.24, suggesting that 24% of the variance in healthcare-seeking is attributable to differences across clusters. This justifies the use of a multilevel approach.

Four models were calculated: model-0 (null model), model-I (which only included variables at the individual level), model-II (which only included variables at the community level), and model-III (which included both individual and community level variables).

The model was specified as:

$${\mathrm{logit(}}\pi_{ij} {) = }\beta_{0j} + \sum\limits_{k} {\beta_{k} X_{kij} }$$

With a random intercept at the community level:

$$\beta_{0j} = \gamma_{00} + \sum\limits_{m} {\gamma_{m} W_{mj} + u_{0j} }$$

The combined model was specified as:

$${\mathrm{logit}}(\pi _{{ij}} ) = \gamma _{{00}} + \sum\limits_{k} {\beta _{k} X_{{kij}} } + \sum\limits_{m} {r_{m} W_{{mj}} + u_{{0j}} }$$

where

πij = P(Yij = 1) is the probability that child i in community j is zero-dose.

Xkij are individual-level predictors (e.g., maternal education, ANC visits).

Wmj are community-level predictors (e.g., residence).

u0j ∼N(0, σu2) is the random intercept for community j.

Yij∼Bernoulli(πij).

## Results

### Socio-demographic characteristics of study participants

The mean age of mothers was 27.40 years (Standard deviation (SD) =  ± 6.01), 9.06% of them were adolescents. Most mothers (94.39%) were currently married, and 43.63% attended primary education. One third (39.44%) of households were classifies as having poor wealth index, and 32.65% had 1–3 members. The mean age of fathers was 33.43 (SD =  ± 11.9), with 60.42% aged 30–45 years, and 39.35% attained primary education, 32.97% of the participant where orthodox 48.62% of the study participant are from Oromia region and 42.08% are from urban (Table 1).

**Table 1:** Weighted Socio-demographic characteristics of the study participants: Evidence from PMA Cohort 2 (2021–2023) in Four Ethiopian Regions.

	Frequency	Percent
Residency		
Urban	348.00	42.08
Rural	479.00	57.92
Region		
Amhara	188.59	22.96
Oromia	399.44	48.62
SNNP	196.06	23.87
Addis	37.40	4.55
Religion		
Orthodox	270.87	32.97
Muslim	299.11	36.41
Protestant	239.99	29.21
Others	11.53	1.40
Father age		
18–29	246.43	30.84
30–45	482.83	60.42
> 45	69.84	8.74
Mother age		
15–19	89.43	10.89
20–24	180.10	21.92
25–29	262.08	31.90
30–34	169.05	20.58
35–39	90.75	11.05
40–49	30.08	3.66
Mother education		
Never attended	272.91	33.22
Primary	358.46	43.64
Secondary	119.30	14.52
Above secondary	70.82	8.62
Father education		
Never attended	222.91	28.02
Primary	339.12	42.62
Secondary	130.78	16.44
Above secondary	102.83	12.92
Marital status		
Currently married	776.64	94.54
Currently not	44.86	5.46
Family size		
1–3 Person	268.30	32.66
4–6 Person	389.41	47.40
7–13 Persons	163.79	19.94
Wealth quantile		
Poor	324.00	39.44
Middle	158.47	19.29
Rich	339.03	41.27

### Reproductive and child health related factors

**Fig. 1:**
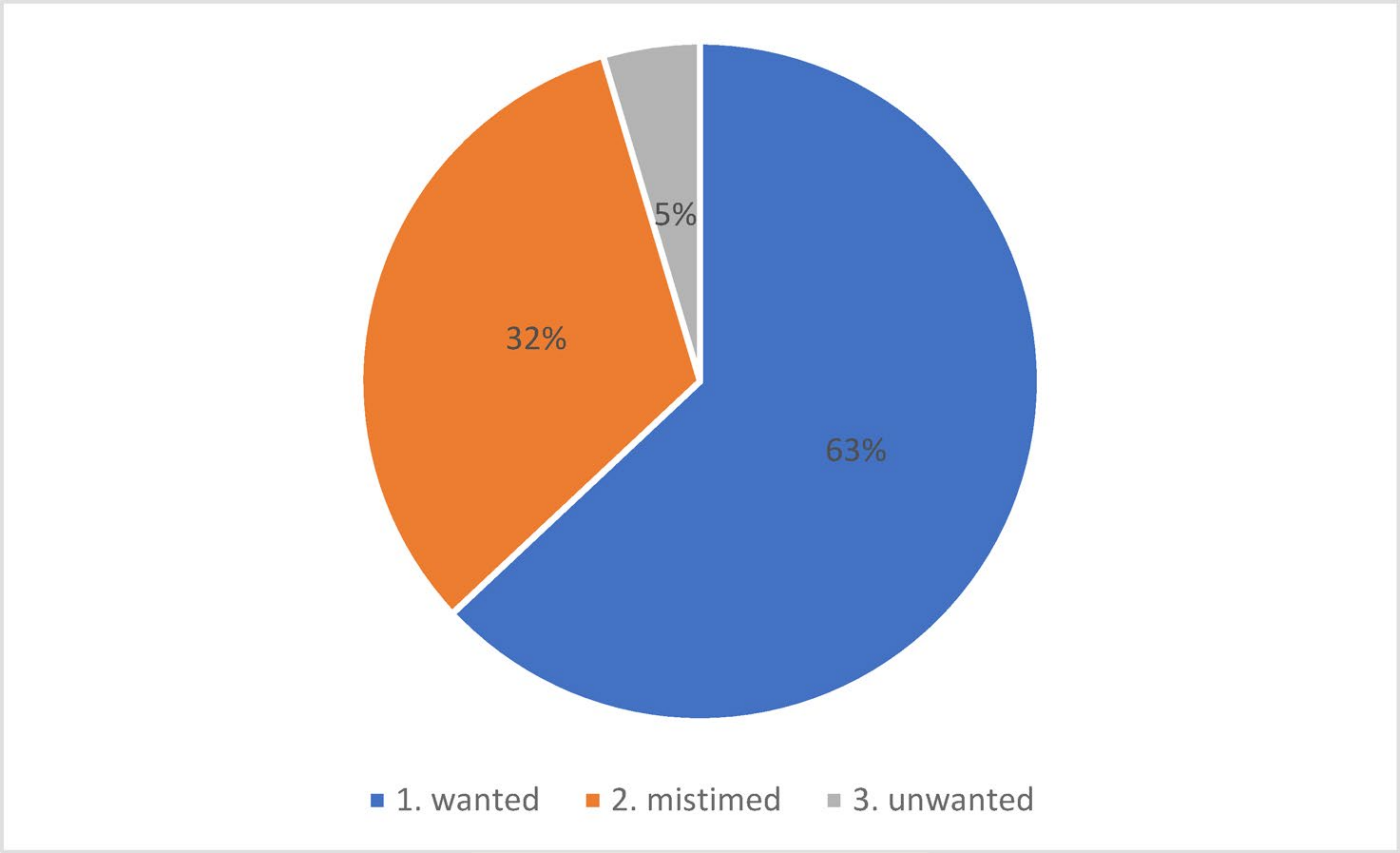
Distribution of recent pregnancy intention: evidence from PMA Cohort 2 (2021–2023) in Four Ethiopian Regions.

98.84% of deliveries was singleton, 70.23% of mothers attended ANC by skilled health professional for the last pregnancy, 63.91% delivered in health facility, 5.7% had PNC visit and 44.56% used FP at least one time. 107.3 (15.71%) mothers reported having physical violence, 171.68 (24.91%). Majority of mothers recent pregnancy (63%) were wanted and 40.29% of women were categorized as empowered in using FP and (46.38%) have respectful maternity care (Table 2, and Fig. 1).

**Table 2:** Weighted Reproductive and child health related factors of the study participants: Evidence from PMA Cohort 2 (2021–2023) in Four Ethiopian Regions.

	Category	Frequency	Percent
Type of pregnancy	Singleton	811.99	98.84
	Twins	9.51	1.16
Physical IPV	No	575.5	84.29
	Yes	107.3	15.71
Sexual IPV	No	517.47	75.09
	Yes	171.68	24.91
IPV any type	No	468.90	67.39
	Yes	226.93	32.61
ANC by health extension worker	No	600.93	73.48
	Yes	216.91	26.52
ANC by skilled health professional	No	242.87	29.77
	Yes	573.05	70.23
PNC at 6 weeks by health extension worker	No	741.86	94.29
	Yes	44.91	5.71
FP use ever	No	455.45	55.44
	Yes	366.04	44.56
Place of delivery	Home	294.98	36.08
	Health institution	522.57	63.92
Women empowerment	not empowered	490.51	59.71
	Empowered	330.99	40.29
Respectful maternity care	Lower RMC)	250.51	53.62
	Higher RMC	216.68	46.38

Health institution includes government hospitals, government health centers, government health posts, NGO/faith-based facilities, and private hospitals/clinics.

### Community level factors

Nearly three fourth (74.45%) of participants were from rural areas, 4.553% were from Addis Ababa city, 67.46% were from low proportion of educated and 62.78% were from high proportion of rich (Table 3).

**Table 3:** Weighted community level factors of the study participants: Evidence from PMA Cohort 2 (2021–2023) in Four Ethiopian Regions.

Region	Frequency	Percent
Amhara	188.59	22.96
Oromia	399.44	48.62
SNNP	196.06	23.87
Addis Abeba	37.40	4.55
Mothers’ literacy		
Low proportion of educated	555.35	67.60
High proportion of educated	266.15	32.40
Place of residency		
Low proportion of urban	611.65	74.46
High proportion of urban	209.85	25.55
Level of wealth		
Low proportion of rich	305.77	37.22
High proportion of rich	515.73	62.78

SNNP refers to the former Southern Nations, Nationalities and Peoples’ Region classification used in the PMA-Ethiopia dataset sampling frame. In this study, it includes only areas currently recognized as South Ethiopia, Central Ethiopia, and Southwest Ethiopia regions; Sidama region was not included in the sample.

### Prevalence of healthcare-seeking behavior among caregiver for common childhood illness

The weighted prevalence of health-seeking behavior for common childhood illness among caregivers where was 39.6% (95% CI 35.8–43.3%).

**Fig. 2:**
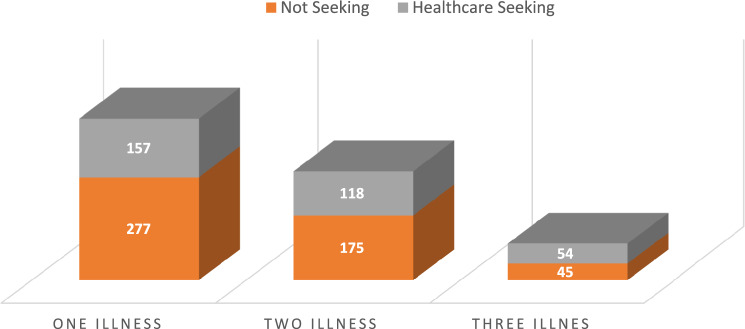
Distribution of health seeking behavior by number of symptoms: evidence from PMA Cohort 2 (2021–2023) in Four Ethiopian Regions.

Among children who experienced illness within the two weeks preceding the survey, the proportion of caregivers who sought healthcare increased with the number of reported illnesses. Specifically, 36.3% of those with one illness sought healthcare, compared with 40.4% among those with two illnesses and 54.6% among those with three illnesses (Fig. 2).

**Fig. 3:**
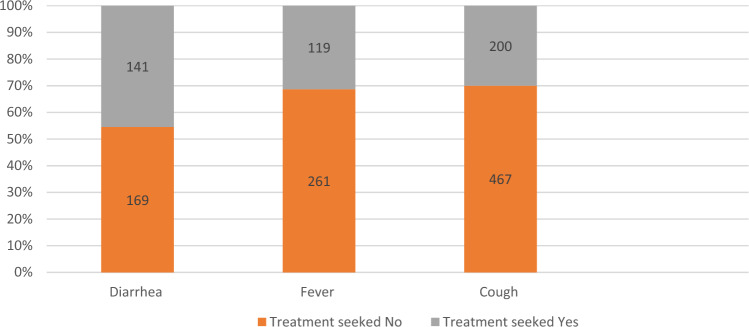
Distribution of health seeking behavior by types of symptoms: evidence from PMA Cohort 2 (2021–2023) in four Ethiopian Regions.

Treatment-seeking rates differed by symptom: 45.5% of caregivers sought care for children with diarrhea, compared to 31.3% for those with fever and 30.0% for those with a cough (Fig. 3).

### Factors associated with healthcare-seeking behavior among caregiver for common childhood illness

#### Random effects analysis

The results of the random-effects analysis are presented in Table  4. In the null model (M0), which contained no explanatory variables, the community-level variance of healthcare-seeking behavior among caregiver for common childhood illness was estimated at 1.04. The intra-class correlation coefficient (ICC) indicated that 24% of the total variation in healthcare-seeking behavior was attributable to differences between clusters. The median odds ratio (MOR) was 2.64, suggesting considerable heterogeneity across clusters.

**Table 4:** Random effect analysis.

Model	Variance	ICC	MOR	-2LogL	PCV
M0 (Null)	1.04	0.24	2.64	1053.82	
M1 (Individual)	1.08	0.25	2.69	1004.27	-3.83
M2 (Contextual)	0.98	0.23	2.57	1049.28	5.39
M3 (Full)	0.97	0.23	2.56	997.47	6.16

When individual-level covariates were included in Model 1 (M1), the cluster variance slightly increased to 1.08, with an ICC of 24.6% and an MOR of 2.69. This model showed a deviance reduction to 1004.8 compared with the null model, although the proportional change in variance (PCV) was negative (–3.83%), indicating that individual-level factors alone did not explain the between-cluster variation.

Model 2 (M2), which included only contextual-level variables, showed a reduction in community variance to 0.98, corresponding to an ICC of 23% and an MOR of 2.57. The PCV was 5.39%, indicating that contextual factors explained a notable portion of the variance between clusters.

In the full model (M3), which incorporated both individual- and contextual-level variables, the community-level variance was further reduced to 0.97. The ICC decreased to 22.8% with an MOR of 2.56, while the deviance was minimized (997.47), suggesting the best model fit. The PCV in the full model was 6.16%, indicating that the combined effect of individual and contextual variables explained part of the between-cluster variance.

Overall, the random-effects estimates demonstrate significant clustering of healthcare-seeking behavior at the community level. The contextual model explained the largest proportion of variance reduction, while the full model provided the best overall fit (Table  4).

### The fixed effect analysis result

In the final multivariable model, antenatal care (ANC), place of delivery, number of illnesses reported in the two weeks preceding the survey, and household wealth status were significantly associated with caregivers’ healthcare-seeking behavior for under-five children with common childhood illnesses.

Mothers who received ANC from health extension workers were 46% less likely to seek health care for their sick children compared to those who did not (AOR = 0.54; 95% CI 0.33–0.88). In contrast, mothers who delivered their child in a health facility were 1.64 times more likely to seek care for childhood illnesses compared to those who delivered at home (AOR = 1.64; 95% CI 1.01–2.69).

The likelihood of seeking healthcare also increased with the number of recent illnesses. Caregivers whose children had two illnesses within the two weeks prior to data collection were more than three times as likely to seek care compared to those whose child had only one illness (AOR = 3.40; 95% CI 1.92–6.01). Similarly, caregivers of children with three illnesses were 1.55 times more likely to seek care (AOR = 1.55; 95% CI 1.06–2.07).

Household economic status was also an important predictor. Caregivers from richer households were 2.10 times more likely to seek healthcare for their children’s illnesses compared with those from poorer households (AOR = 2.10; 95% CI 1.09–4.04) (Table 5).

**Table 5:** Multilevel multivariable logistic regression analysis of factors associated with caregivers’ health care seeking behavior for common childhood illness : Evidence from PMA Cohort 2 (2021–2023) in Four Ethiopian Regions.

Individual level factors	Null Model	M1 (Individual)	M2(Contextual)	M3(Full)
		AOR (95% CI)	AOR (95% CI)	AOR (95% CI)
(Intercept)				
ANC by health extension worker				
No		1.00		1.00
**Yes**		**0.57(0.35,0.92)**		0.54(0.33,0.88) **
Place of delivery				
Home		1.00		1.00
**Health institution**		**1.50(0.925,2.)**		1.64(1.01,2.69) **
Age of the mother				
Age 15–19 years		1.00		1.00
Age 20–24 years		0.68(0.34,1.345)		0.72(0.36,1.44)
Age 25–29 years		0.93(0.46,1.90)		0.98(0.48,2.00)
Age 30–34 years		1.01(0.45,2.23)		1.08(0.49,2.40)
Age 35–39 years		0.71(0.29,1.76)		0.79(0.32,1.96)
Age 40–49 years		1.45(0.38,5.60)		1.65(0.43,6.31)
Women empowerment				
Not empowered		1.00		1.00
Empowered		1.14(0.77,1.70)		1.15(0.77,1.70)
Family size				
1–3 person		1.00		1.00
4–6 person		1.20(0.79,1.81)		1.18(0.78,1.79)
7–13 persons		1.46(0.80,2.64)		1.37(0.75,2.49)
Ever used family planning				
No		1.00		1.00
Yes		1.26(0.86,1.85)		1.31(0.89,1.93)
Gender of the baby				
Male		1.00		1.00
Female		0.73(0.52,1.03)		0.73(0.52,1.02)
Recent pregnancy				
Wanted		1.00		1.00
Mistimed		1.02(0.63,1.63)		1.02(0.63,1.64)
Unwanted		1.33(0.61,2.88)		1.29(0.59,2.78)
Household level of wealth				
Poor		1.00		1.00
Middle		1.22(0.69,2.14)		1.27(0.72,2.22)
**Rich**		**1.57(0.91,2.71)**		2.10(1.09,4.04) **
Number of illnesses with				
One illness		1.00		1.00
**Two illness**		**3.37(1.90,5.97)**		3.40(1.92,6.01) **
**Three illness**		**1.55(1.06,2.27)**		1.55(1.06,2.27) **
Respectful maternity care				
Low RMC		1.00		1.00
High RMC		1.37(0.82,2.27)		1.39(0.82,2.36)
IPV any				
No		1.00		1.00
Yes		1.09(0.68,1.74)		1.11(0.70,1.77)
Community level factors				
Place of residence				
Low proportion of urban			1.00	1.00
High proportion of urban			0.51(0.93,0.51)	0.49(0.24,1.02)
Region				
Amhara			1.00	1.00
Addis Abeba			0.97(0.40,2.33)	0.96(0.39,2.36)
Oromia			1.41(0.77,2.58)	1.48(0.78,2.81)
SNNP			1.22(0.64,2.34)	1.31(0.66,2.60)
Community level of literacy				
Low proportion of educated			1.00	1.00
High proportion of educated			1.60 (0.91,2.81)	1.29(0.71,2.32)

SNNP refers to the former Southern Nations, Nationalities and Peoples’ Region classification used in the PMA-Ethiopia dataset sampling frame. In this study, it includes only areas currently recognized as South Ethiopia, Central Ethiopia, and Southwest Ethiopia regions; Sidama region was not included in the sample. Significant values are in [bold]

## Discussion

Seeking healthcare for common childhood illnesses is vital for reducing under-five morbidity and mortality. In this study, several individual and household factors were significantly associated with caregivers’ health-seeking behavior, including place of delivery, antenatal care (ANC) by health extension workers, number of childhood illnesses within the two weeks prior to data collection, and household wealth status.

The prevalence of health-seeking behavior for common childhood illnesses in this study 39.6% (95% CI 35.8–43.3%) indicates that fewer than half of caregivers sought care, which is consistent with earlier findings from Ethiopia showing that about 38% (95% CI 34–41%) of caregivers sought care^26^. However, this level is lower than the pooled national estimate of 46.6% (95% CI 38.7–54.4) reported in the 2022 systematic review^13^. Care-seeking was higher for diarrhea (45.5%) than for fever (31.3%) and cough (30.0%), a pattern that aligns with the 2016 EDHS, which reported healthcare-seeking rates of 44.1% for diarrhea, 35.7% for fever, and 29.1% for acute respiratory infection^7^.

Caregivers who delivered in health facilities were more likely to seek health care for their sick children compared to those who delivered at home. This is consistent with findings from Ethiopia, where facility delivery was linked to improved utilization of child health services^21,27^. Similar associations have been reported in Nigeria and other low- and middle-income countries, where facility delivery enhances mothers’ familiarity with formal health systems and provides opportunities for health promotion. A plausible explanation is that women who deliver at facilities gain confidence in health services and receive counseling that encourages future care seeking^28^.

Mothers who received ANC services from health extension workers were less likely to seek health care for their under-five children compared to mothers who did not. This finding is unexpected, as ANC generally provides opportunities for health education and linkage to formal health services. However, similar challenges have been noted in Ethiopia, where the effectiveness of health extension programs varies depending on service quality and counseling content^29^. Evidence from other sub-Saharan African countries also suggests that the quality and comprehensiveness of ANC—not simply its occurrence determine subsequent health service use^30^. One possible explanation is that ANC visits delivered by health extension workers may have focused on maternal rather than child health, leading to insufficient emphasis on timely care seeking for children’s illnesses, and the other reason could be ANC is non-therapeutic follow for healthy pregnancy, whereas child illness needs therapeutic and needs therapeutic mgt by nurses or physicians, but not by HEW. So that women may not seek care from HEWs.

The number of illnesses experienced by a child in the two weeks preceding the survey was also strongly associated with healthcare-seeking behavior. Caregivers whose children had two or more illnesses were more likely to seek care compared to those with only one illness. Although Ethiopian studies rarely disaggregate healthcare-seeking by number of illnesses, evidence indicates that perceived severity and multiplicity of symptoms increase the likelihood of care seeking^8,31^. This suggests that caregivers respond more actively when the burden of illness is greater.

Household wealth was another important determinant of healthcare-seeking behavior. Caregivers from richer households were more likely to seek healthcare for their children compared to those from poorer households. This finding is consistent with national analyses of Ethiopian Demographic and Health Survey data, which demonstrated persistent wealth-related disparities in care seeking for childhood illnesses^7^. Comparable results have been documented in other African settings and in South Asia, where higher economic status is consistently associated with better access to and utilization of health services^32,33^ .A key explanation is that wealthier households face fewer financial barriers, can afford transportation and healthcare costs, and are more exposed to health information through various media outlets.

### Implications

The findings underscore the need for multifaceted interventions. Improving the quality and content of ANC services provided by health extension workers could increase trust and promote timely care-seeking. Strengthening institutional delivery coverage may have spillover benefits on subsequent health-seeking for child illnesses. Economic disparities remain a barrier, suggesting that financial protection mechanisms and community-based support could enhance equitable access. Public health interventions should also raise awareness about the importance of early healthcare, especially for children with mild or single symptoms, to prevent progression to severe illness.

### Strength and limitation

Some limitations should be noted. First, the analysis was based on secondary survey data, which restricted variables to those available in the dataset. Second, caregiver self-reports of childhood illness and care-seeking behavior may be subject to recall or reporting bias. Third the study sample was drawn from only four regions, which may restrict the generalizability of the results to the national population. Finally, the cross-sectional design limits causal inference. Despite these limitations, the study’s robust multilevel modeling provides valuable evidence for policymakers and program planners. Missing data were addressed using multiple imputation techniques, minimizing potential bias and enhancing the validity of the findings. Furthermore, the comprehensive inclusion of socio-demographic, reproductive, and psychosocial factors allowed for a multidimensional understanding of the determinants of health seeking behavior among caregivers for common child hood illness.

## Conclusion

This study demonstrated that healthcare-seeking for common childhood illnesses remains low in Ethiopia, indicating a persistent gap in timely care utilization. Care-seeking behavior was significantly associated with maternal health service experiences, child illness burden, and household wealth. Mothers who received ANC from health extension workers were less likely to seek care for sick children, whereas facility-based delivery, the presence of multiple childhood illnesses, and higher household wealth were positively associated with healthcare utilization. These findings suggest the need to strengthen the quality and counseling components of ANC provided by health extension workers, reinforce continuity between maternal and child health services, and address economic barriers that limit access to care.

## Data Availability

This study is based on publicly available de-identified secondary data from the PMA Ethiopia project. Access was granted freely without registration in john Hopkin research repository. https://archive.data.jhu.edu/dataverse/pmaet The original data collection protocols were approved by the Institutional Review Board (IRB) of Addis Ababa University and Johns Hopkins Bloomberg School of Public Health. No personal identifiers were used in this secondary analysis.

## Abbreviations

ARI: Acute respiratory tract infection
PMA-E: Performance monitoring for action, Ethiopia
SNNPR: Southern nations, nationalities, and peoples’ region
EA: Enumeration area
RMC: Respectful maternity care
ICC: Intra-class correlation
SD: Standard deviation
ANC: Antenatal care
PNC: Postnatal care
FP: Family planning
IPV: Intimate partner violence
MOR: Median odds ratio
PCV: Proportional change in variance
IRB: Institutional review board

## Footnotes

1 SNNP refers to the former Southern Nations, Nationalities and Peoples’ Region classification used in the PMA-Ethiopia dataset sampling frame. In this study, it includes only areas currently recognized as South Ethiopia, Central Ethiopia, and Southwest Ethiopia regions; Sidama region was not included in the sample.
